# Supplementary material for: Self-Hybridized Exciton-Polaritons in Sub-10-nm-Thick WS2 Flakes: Roles of Optical Phase Shifts at WS2/Au Interfaces
Source: Nanomaterials (Basel). 2022 Jul 13;12(14):2388. doi: 10.3390/nano12142388 (PMC9319842; doi:10.3390/nano12142388)
Supplement: Supplementary file 1 [file nanomaterials-12-02388-s001.zip › nanomaterials-1746686-supplementary.pdf]

nanomaterials

MDPI

Article

Self-Hybridized Exciton-Polaritons in Sub-10-nm-Thick WS_2 Flakes: Roles of Optical Phase Shifts at WS_2/Au Interfaces

Anh Thi Nguyen, Soyeong Kwon, Jungeun Song, Eunseo Cho, Hyohyeon Kim and Dong-Wook Kim *

Department of Physics, Ewha Womans University, Seoul 03760, Korea; nthianh111@gmail.com (A.T.N.); kwonsso91@gmail.com (S.K.); sje10056996@gmail.com (J.S.); escho797@gmail.com (E.C.); kimhyohyun1614@gmail.com (H.K.)

* Correspondence: dwkim@ewha.ac.kr

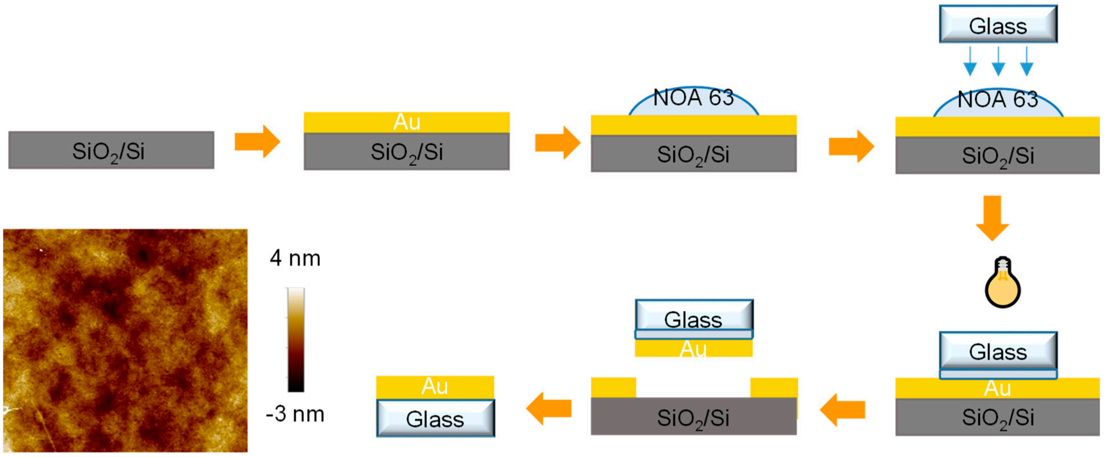

Figure S1. Schematic diagrams to illustrate the process to prepare template-stripped Au layers. UV-curable pre-polymer (NOA 63, Norland) was used to delaminate the Au thin films deposited on SiO_2/Si template substrates. A typical root-mean-square roughness of the Au layer was 0.7 nm, as shown an exemplary atomic force microscopy image (area: 10×10 μm²).

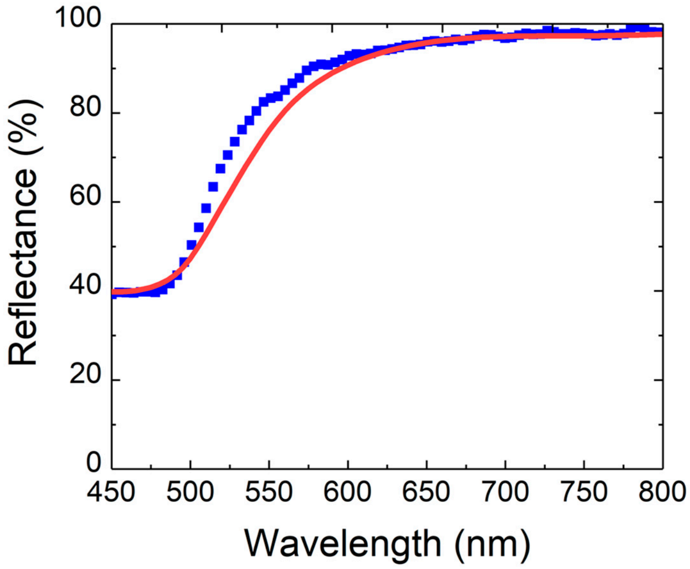

Figure S2. Typical measured reflectance spectrum of our template-stripped Au layer (blue squares) and TMM-calculated spectrum of a flat Au thin film (red solid line). The reflectance spectrum of the Au layer does not show any feature originated from the surface plasmonic effects. The measured spectrum is very similar to the spectrum of an ideal flat Au thin film obtained by the transfer matrix method.

nanomaterials

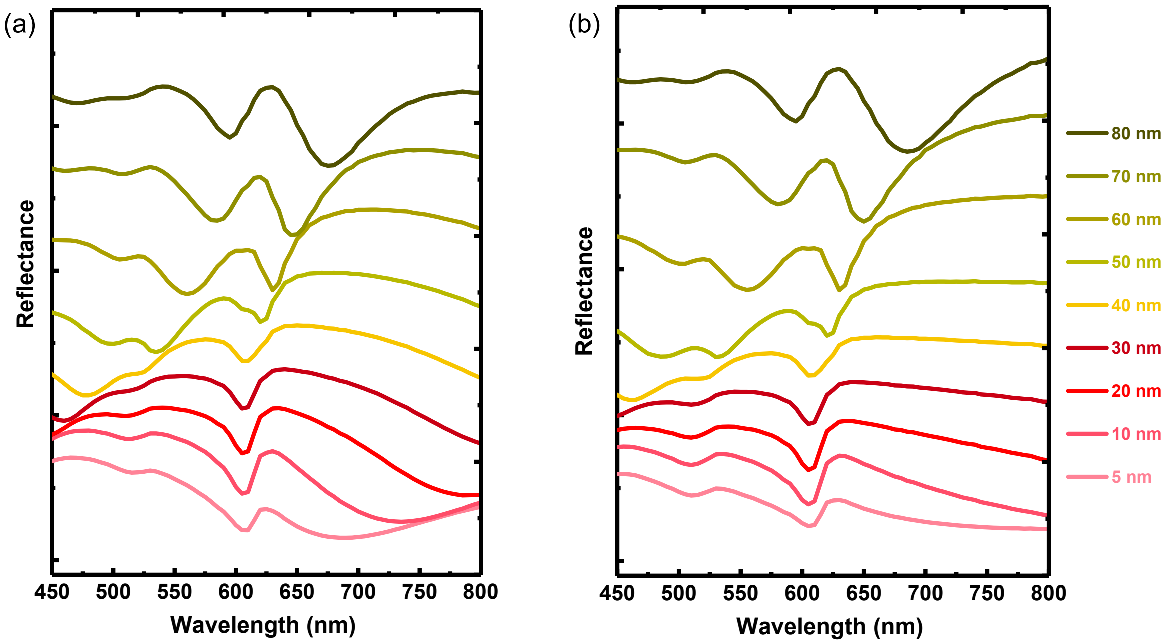

Figure S3. TMM-calculated thickness-dependent reflectance spectra of WS_2 flakes on (a) 300- and (b) 90-nm-thick SiO_2/Si substrates. Comparison of the spectra shows that the thin film interference in the substrates affects the spectral responses of $WS_2/SiO_2/Si$ at long wavelengths.

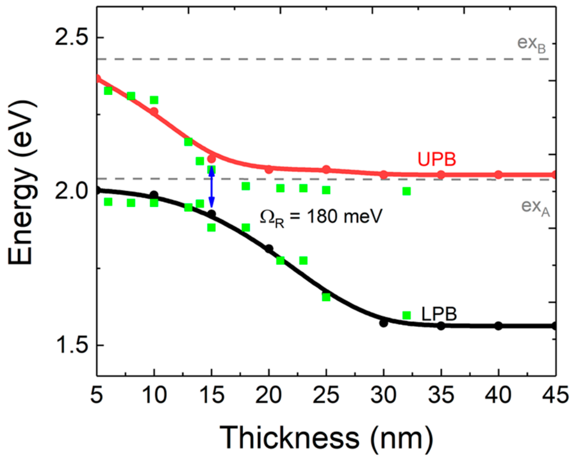

Figure S4. Thickness-dependent UPB- and LPB-energies of WS_2 flakes with d_{WS2} < 50 nm on Au. Green squares indicate the measured data in Fig. 3a of the manuscript. Solid lines with circles correspond to the TMM calculation results (Figs. 2c and 3b of the manuscript). The Rabi splitting energy for the A exciton in WS_2 flakes can be estimated to be ~180 meV.
